# Parents’ Experiences With Ultrasound During Pregnancy With a Lethal Fetal Diagnosis

**DOI:** 10.1177/2333393615587888

**Published:** 2015-06-01

**Authors:** Erin M. Denney-Koelsch, Denise Côté-Arsenault, Erin Lemcke-Berno

**Affiliations:** 1 University of Rochester, Rochester, New York, USA; 2 The University of North Carolina at Greensboro, Greensboro, North Carolina, USA

**Keywords:** communication, medical, naturalistic inquiry, illness and disease, experiences, illness and disease, life-threatening / terminal, pregnancy, high-risk

## Abstract

This longitudinal naturalistic study sought to describe parent experiences of ultrasounds during pregnancies with lethal fetal diagnoses (LFDs). We interviewed 16 mothers and 14 partners twice during pregnancy and twice after birth and death of their infant. Parents reported that ultrasound providers had a profound impact on their experiences with LFDs. Within three stages of pregnancy (pre-diagnosis, learning the diagnosis, and living with the diagnosis), themes of *optimistic expectation, hearing bad news, need to know*, and *time with baby* emerged. The dynamics of interactions with ultrasound providers included *differing goals and expectations*, and *compatibility of interactions*. These interactions were either satisfying or added to parents’ burden. Ultrasound providers have the opportunity to share valuable knowledge and facilitate understanding and precious time with the baby. Providers of obstetrical care can improve communication with parents with LFDs at critical time periods by matching their interaction to parents’ needs.

The use of prenatal ultrasound for screening during pregnancy has become widespread. Obstetricians recommend mid-trimester screening ultrasounds for all pregnant women (American College of Obstetrics and Gynecology, 2009; American Institute of Ultrasound in Medicine, 2013). Parents often eagerly anticipate their ultrasounds and see them as a joyful part of the pregnancy experience. Ultrasound examinations often reduce anxiety and increase prenatal attachment in the pregnancy (Ekelin et al., 2009; Lalor & Begley, 2006). However, in the 2% of pregnancies in which serious fetal abnormalities are found (Levi, 2002), it is frightening and traumatic to learn that one’s fetus has a lethal condition (Aite et al., 2011; Skreden et al., 2010). Parents often experience anxiety, depression, and intense grief reactions (Côté-Arsenault & Denney-Koelsch, 2011). When an abnormality is detected, families have reported awkward interactions with sonographers: He or she may suddenly go quiet, spend a lot of time going over one area, or turn the screen away from the patient (Côté-Arsenault & Denney-Koelsch, 2011).

Obstetrical sonographers are unique among imaging technologists, because they routinely have extended interactions with patients and the potential to share results in real time, yet sonographers are rarely formally trained in patient communication or counseling (Commission on Accreditation of Allied Health Education Programs, 2011). A sonographer’s response to a finding of a lethal anomaly may vary, according to his or her professional experience and the policies and procedures established by the interpreting physicians. Some sonographers are required to wait for the physician to explain the findings. Sonographers in the United Kingdom identified multiple complexities in relaying bad news to patients, and found these interactions easier if an established protocol in their institutions was established (Simpson & Bor, 2001).

Some evidence suggests that patients receiving standard ultrasounds prefer receiving results immediately from their sonographer, rather than waiting for the referring physician (Ragavendra, Laifer-Narin, Melany, & Grant, 1997), but a Cochrane Review of feedback during ultrasounds with normal pregnancies indicated a lack of sufficient evidence that receiving immediate feedback from the sonographer reduces stress (Nabhan & Faris, 2010). Survey results have shown that women with fetal anomalies value prompt provision of high-quality information, given by a sympathetic and well-informed provider (Alkazaleh et al., 2004; Lalor, Devane, & Begley, 2007). They want timely referrals to experts and find written information helpful (Nabhan & Faris, 2010). Women in Canada with a history of pregnancy loss were keenly aware of the sonographer’s behavior including nonverbal signals, room layout, and placement of the ultrasound screen (O’Leary, 2005; Van der Zalm & Byrne, 2005). No known U.S. studies have explored what parents need from their sonographer at the moment when a potentially lethal condition is discovered or at subsequent ultrasounds.

Because both physicians and sonographers sometimes do ultrasounds, we will refer to both as “ultrasound providers.” Although the health care communication literature focuses mainly on physician–patient interactions, encounters with other health care providers share many of the same characteristics. Any health care provider who is the holder of knowledge and diagnostic tools is a key source of information for patients. The patient likely begins the interaction with greater needs and higher stakes than the provider, leading to a power differential (Ha, 2010; Ishikawa, Hashimoto, & Kiuchi, 2013). Satisfaction with interactions, from the perspectives of both provider and patient, is enhanced when this asymmetry is minimized by good communication skills and a collaborative, patient-centered approach. The provider empowers the patient with knowledge and facilitates shared decision making (Ha, 2010; Ishikawa et al., 2013). There is little literature, guidelines, or training programs to guide ultrasound providers in the care of parents with lethal fetal diagnoses (LFDs).

The purpose of this article is to describe parent experiences of ultrasound examinations when lethal anomalies were detected and to examine their interactions with ultrasound providers at multiple time points during the pregnancy. This study is part of a larger qualitative, longitudinal study describing the overall experience of pregnancy with LFDs and what parents need during interactions with their health care providers.

## Method

We conducted a longitudinal, naturalistic study, using convenience and purposive sampling, of parents’ experiences of continuing a pregnancy with a LFD.

We recruited women if they were 18 years or older, pregnant, English speaking, and had decided to continue their pregnancy after learning of a LFD (defined as estimated neonatal prognosis of 2 months or less), excluding multiple gestations. Partners or spouses were asked to participate by women who chose to be in the study.

Genetics counselors, obstetricians, neonatologists, palliative care physicians, nurses, doulas, and community support groups purposively sampled potentially eligible participants at five conveniently accessible institutions in upstate New York, North Carolina, and Wisconsin. These care providers asked for parents’ permission prior to referring them to the study. Human Subjects Review Board approval was obtained from both of the authors’ institutions. Informed consent was obtained from each participant.

### Data Collection

We performed the first interview as close to the time of fetal diagnosis as possible. We interviewed mothers and spouses/partners together to hear their collective story and then individually, whenever possible, to hear their unique, individual experiences. Our aim was to conduct two prenatal interviews (one in the second trimester and one in the third trimester), and two after the birth and death of the infant. In-depth interviews took place either in person, by phone, or on a video conference (Skype™), based on parent preference and geographic feasibility. Interviews lasted 30 minutes to 2 hours. Participants were reminded that the interview could be paused at any point, and were provided a resource list for further emotional support if needed. We reimbursed US$20 per person per interview for their time and effort, and gave a small gift after the first and last interviews.

The principal investigator (PI; D.C.) conducted all interviews by using an interview guide that began with “Tell me about this pregnancy, from the beginning,” and also, “Could you tell me about your visits with doctors, nurses, sonographers, specialists? Tell me about what is important to you, right now in your pregnancy.” The focus of the interviews evolved over time to match the gestational age of pregnancy, to clarify concepts and experiences identified during analysis, and changed after birth to issues of bereavement and memory making. Data collection ended when stories became redundant.

We digitally recorded, professionally transcribed, and carefully verified all transcripts. Field notes were written by the PI after each interview to capture the nuances and tone of each interview and each participant; nonverbal behaviors were inserted into the final transcript of in-person and teleconference interviews.

### Data Analysis

We de-identified all transcripts and imported them into Atlas.ti7 for data management. The research team consisted of a nurse researcher (PI), physician (co-investigator), project coordinator, and sonographer. Data analysis began during data collection and continued through transcript verification, two cycles of coding, theme identification, within- and cross-case analysis, and the writing of results (Miles, Huberman, & Saldana, 2014). During data analysis of our larger study on parents’ experiences of pregnancy with LFDs, themes related to the profound impact of ultrasounds on parents’ experiences emerged, leading to this focused report. We pulled codes related to ultrasound, sonographer, or sonographer interaction, identified themes, and discussed them in team meetings. We performed within-case analysis as each interview was conducted and coded. Team members wrote memos throughout data analysis and during coding and research team discussions, whenever patterns in the data were identified. Team members compiled matrices to display case-by-case chronology to facilitate cross-case comparisons. Cross-case analysis within and across couples occurred through constant comparison.

Trustworthiness of this study was promoted by using a single interviewer, having a team of analysts including a sonographer, identifying all related quotes using the Atlas.ti query function, and reaching consensus on our themes within our research team.

## Results

### Sample

A sample of 16 mothers, 13 fathers, and 1 lesbian partner enrolled in the study. Demographics, obstetrical history, and fetal diagnoses are displayed in Tables 1 and 2. Subjects resided in four mid-Atlantic and upper mid-western states and received care at a variety of institutions, ranging from large university hospitals to community hospitals. First interviews were conducted at 20 to 35 weeks of gestation. One couple was lost to follow-up after the first interview. The remaining 15 participated in three to five interviews each for a total of 90 interviews. All initial ultrasounds were done at private obstetrics or family medicine offices; subsequent ones were all done at perinatal referral centers. No differences were found within couple reactions to ultrasound experiences.

**Table 1.** Demographic and Obstetrical History of the Sample.

	Mothers	Fathers/Partners
	*n* = 16	*n* = 13/1
Age		
Range, *M* (*SD*)	22–42, 32.9 (5.5)	21–49, 33.64 (7.2)
Race *n* (%)		
Caucasian	11 (68.8%)	10 (62.5%)
African American	3 (18.8%)	2 (12.5%)
Hispanic	1 (6.3%)	2 (12.5%)
Asian/Pacific Islander	1 (6.3%)	0
Education in years		
Range, *M* (*SD*)	12–21, 15.1 (2.8)	10–19, 13.9 (2.9)
Family income		
Range (median)	0 to >120,000 (US$60–US$80,000)	
No. of pregnancies		
Range, *M* (*SD*)	1–8, 2.6 (2.0)	
History of infertility (*n*)	3	
History of miscarriage (*n*)	3	
No. of living children		
Range, *M* (*SD*)	0–7, 1.5 (1.9)	

**Table 2.** Fetal Diagnoses in the Sample.

Fetal Diagnosis	*n*
Anencephaly	1
Ectopia cordis	1
Partial trisomy 5p	1
Severe oligohydramnios	3
Severe skeletal dysplasia	1
Tetrasomy 9p	1
Trisomy 13	4
Trisomy 18	4

### Profound Impact

The overall impression of sonographer–parent interactions during obstetrical ultrasounds was that they had a profound impact on parents in our sample, all of whom heard and were coping with the devastating news. As one mother commented, “That’s where it all begins.” At the ultrasound, the pregnancy journey abruptly changed its focus. Sonographers provided the parents with access to critical information and a treasured opportunity to see their baby. We were struck by the spectrum of parents’ responses to each ultrasound, ranging from highly satisfied to angry and frustrated. The interaction either met their needs or added to their burden. Parents nearly always looked forward to their ultrasounds, even after the lethal diagnosis: They provided a way to see and connect with their baby.

### Pregnancy Stages and Changing Needs

We identified three distinct stages during the pregnancy where ultrasound interactions were reported by these parents: pre-diagnosis, learning the diagnosis, and living with the diagnosis. Parents’ needs changed through these stages of pregnancy and the diagnostic process unfolded. At their first ultrasound, parents needed information, and then confirmation and clarification of the diagnosis. Thereafter, they saw the ultrasound as a time to monitor the pregnancy and baby, but more importantly, as a precious and fleeting opportunity to watch and get to know their baby.

### Pre-Diagnosis Stage

#### Optimistic expectation

Whereas for the sonographer, the purpose of the screening ultrasound was to evaluate maternal and fetal structures and rule out abnormalities, parents expected to hear the confirmation of normality and good news. Their expectation of a first-trimester ultrasound was that it would be “routine,” to see the baby’s heartbeat. As one first-time mother stated, “I was expecting everything to be normal.” In the second trimester, they expected to learn their baby’s gender and have a chance to see their baby. Although they were aware that things went wrong with some pregnancies, they did not expect anything to be wrong with their own.

#### Hearing bad news

Learning about an abnormality was a life-altering stage in their experience, a profound crisis. The news occasionally came from a screening blood test, but more often via ultrasound. During the ultrasound, parents were immediately aware of a problem because of the sonographer’s behavior. One couple reported, “We were told something’s not normal . . . my fluid was low . . . but just wait until your next appointment.” In all cases, they experienced a subsequent specialized ultrasound to clarify details.

Parents with prior ultrasound experiences compared this situation with their past scans. A mother with a history of successful and unsuccessful pregnancies felt that “Things were a little different. I’m not real sure what it was, but we were just there to look at, find the sex of the baby that time, and we were not expecting . . . something odd, but something was different.”

In all cases, mothers were referred to a maternal–fetal medicine clinic for further evaluation with additional ultrasounds. Some had to wait up to a week to be seen, which was extremely stressful. One mother who had to wait a week said, “It was torture. It was terrible because . . . I don’t think I slept any. I think we lived in hell that whole time period. It was terrible. It was terrible.”

### Learning the Diagnosis Stage

Learning the diagnosis was a process. Parents first needed a name for what was wrong with their baby, and became better informed as time and details progressed.

#### Need to know

Parents were anxious to see specialists and get more information but also had to brace themselves for the emotional impact of new details. As soon as possible, parents searched for information on the Internet to get a sense of what might be wrong with their baby. One father described his wife looking over the sonographer’s shoulder as she typed because they had been told nothing. As soon as they were done, the mother got on her phone while walking to the obstetrician’s office; she was “looking up ‘strawberry-shaped head.’” Not knowing was extremely stressful. In some cases, a diagnosis was not clear. They found it difficult to live with diagnostic uncertainty.

Each ultrasound brought new expectations, anxiety, and questions. Some sonographers provided extensive details and explanations during specialized ultrasounds. Parents found the facts difficult to hear but were grateful for the information, preferring providers who gave straightforward information, accompanied by pamphlets and written medical terms.

### Living With the Diagnosis Stage

After the diagnosis, all but one couple went for a series of follow-up ultrasounds. Medically they were done to monitor the pregnancy and fetus, and parents wanted to know about any changes or updates they could get. Most parents looked forward to upcoming scans because they provided an opportunity to see their baby. This was particularly meaningful for the non-pregnant parent, who did not have the direct physical experience of carrying the baby.

#### Checking-in

The first clinical goal during later ultrasounds was monitoring fetal growth and the status of abnormalities (e.g., omphalocele, low amniotic fluid), but parents also looked for any hints of hope. They described positive interactions with providers who spent time, shared details, and knew their situation. Negative interactions were with providers who did not know them, were impersonal, or rushed. One mother shared, “I saw a new doctor. He just kind of didn’t seem like he knew the whole situation and he kind of made it a lot more stressful than it needed to be.” Parents felt cheated when their sense of the importance of the ultrasound was not reflected in the care of the sonographer. One mother expressed her thoughts on a rushed ultrasound:

Well, this is the most important thing to me and you took 10 minutes to do something that I know should take a half an hour. It’s not important to you, but it’s breaking my heart when you’re saying that you can find only one centimeter of fluid. I’m telling you I’m upset about this. Don’t make excuses. At least, validate that I have these feelings!

#### Time with baby

In addition to assessing the baby’s condition, most parents felt that the follow-up ultrasounds were an opportunity to see the baby, get to know the baby, and bond while their child was alive in utero. One mother expressed that “I would’ve liked it to, I guess, done it more just because if he does pass away, I would like to have those memories of him.”

The parent who was not carrying the baby particularly treasured the ultrasounds. One father thoughtfully stated, “I don’t know that it really changes it so much, but I think it’s really wonderful . . . this may be the only opportunity that we actually have to meet her in all her life.” Another father shared that he would have paid for extra ultrasounds had he needed to because “it is nice especially for like me because I don’t get to feel her every day. I don’t have that bond and so to see her is huge for me because I need that.”

One mother brought her teenage son to the ultrasound, as a way to provide an opportunity to meet his sister: “That’s why I made him go with me to the ultrasound because I wanted him to see her in action. I didn’t want him to just get that picture of her [ultrasound photo] in case she didn’t make it. He wants to be there.” She went on to describe them laughing together when he poked his sister through the mother’s abdomen and the baby responded, “It was wonderful!”

Some of the mothers found follow-up ultrasounds to be bittersweet. They enjoyed seeing the baby, but also found it painful. One mother vomited every time she had one. “I like seeing him, but I cry automatically . . . I can’t smile because my heart is so broken.” One mother was particularly bothered by going to follow-up ultrasounds where the sonographer reviewed all the baby’s anomalies in detail every time, and so the mother was not given the opportunity to enjoy the normal aspects of her baby. “I cry at every one because . . . What they see, what I feel when I look at her and what I feel when I’m not there, those are three different things.”

### Dynamics of Interactions

Two dynamics of ultrasound interactions emerged that laid the foundation for parental satisfaction or added burden: differing goals and expectations, and compatibility of interactions.

#### Differing goals and expectations

The participants in these interactions, parents and the sonographer, each had his or her own goals and expectations for each encounter. The sonographer had ultrasound protocols to follow and may be constrained by one’s scope of practice; the purpose of the ultrasound was to provide the physicians with updated information. However, the parents’ focus was to see their baby. They saw the sonographer as the holder of information.

Parents felt satisfied if the sonographer was sensitive to their expectations when delivering information. They also appreciated being given some idea what to expect at the ultrasound: “When I met with the sonographer first and she took about an hour measuring everything with the baby. She’s like, ‘It’s going to take a little long to do this.’ I said, ‘That’s fine.’”

The parents wanted sonographers to be very open with them about what they were seeing. A father pondered on a specialized ultrasound: “Looking back, you know, I left there thinking, ‘I should’ve asked this . . . I should’ve asked that.’ My advice for anybody is try and think as much as you can.”

Parents always wanted the sonographer to know their history prior to entering the room, to eliminate the burden of having to explain. One mother who knew her son’s trisomy diagnosis recalled,

I’d never met her before. I was already a mess just because this was all still so new and I was just scared to death about what was going to happen. She came in and . . . after a few minutes, her first question was, “Have you had a fetal MRI?” [sigh] And I realized that she didn’t know anything . . . And, and then she asked, a few minutes later, “Have you had an amnio?” And I, at that point, I was sobbing . . . I mean I was not just in tears. I was sobbing. And what I really wanted, I wanted her to stop.

Although these parents wanted as much information as possible, they did not want the sonographer to give recommendations about treatments:

He was great and he was very knowledgeable. He kind of gave his opinion of things. The one opinion I don’t think like should be given, he said, “When you have all of the signs that you have, most people terminate” . . . I don’t think I talked to him anymore.

These parents felt that this sonographer had crossed a line.

#### Compatibility of interactions

During the ultrasounds, parents read cues, both verbal and nonverbal, from the sonographer. One father explained: “It just seemed like she took more time. She’s more compassionate. It’s just the way she talked . . . it’s the tone in her voice.” Interactions ranged across a spectrum of styles from silence, a sudden change in tone, to fully engaged.

##### Silence

Parents perceived silence as meaning that either something was wrong or the sonographer was not permitted to tell them any information. If parents perceived that the sonographer was not permitted to reveal information, they accepted that. One mother said, “I don’t think they can legally say anything.” It was perceived as a neutral interaction, one that was neither satisfying, nor did it add to the parents’ burden. Others were hurt and angry, feeling that the sonographer was intentionally withholding important information. Another mother said, “She was horrible . . . This is what she does, it’s her specialty. So I think obviously she knew that it was a significant concern.” This mother wanted to be kept informed:

I think that for me, I’m very much the kind that I want to know what’s going on. I ask questions through the ultrasound: What are you looking at? What am I seeing? What’s that? I did that with my first pregnancies as well. The fact that I couldn’t see that first ultrasound and it was like silent, I mean that was very uncomfortable.

##### Change in tone

Parents sensed a sudden change in the tone, from conversational to silent. One mother describes, “we was in there laughing and joking because like I said, this little girl has a personality that won’t wait . . . Then the nurse got quiet and she was like, ‘Are you sure that your period was when you said?’” This silence led to alarm, especially in this father:

For me, the thing was just from the minute when I could see and they told us it’s a boy, from being up right there [held hand up to show high emotions] to all of a sudden her demeanor changing and realizing something is wrong, that drop in emotion. It’s something I never ever want to go through again in my life . . . You know, you can immediately tell—it became heavy in the room. I knew something was wrong.

Others were frustrated when they had to wait for a physician to explain what was wrong. One couple was particularly upset when they did not sense a problem until the physician’s arrival after the ultrasound.

##### Engaged

Parents were most satisfied when sonographers were highly engaged, showing them the anatomy, explaining the concerns, and spending time showing the normal and abnormal parts of the baby. A father described,

a great ultrasound technician, wonderful guy. He was just—He was very straightforward. There was no silence . . . He spoke to us through everything and he just made us feel, “Listen, you guys, I’m with you through it.” He’s told us everything.

Another physician encouraged parents to advocate for themselves:

She basically said it’s your baby. If you want to see the heart, you see the heart . . . she went over like everything she was doing and everything like that . . . I just remember feeling really comfortable with her because she really talked us through what she was doing.

Parents preferred to be kept informed to the highest degree possible at all of their ultrasounds, from the first moment of diagnosis, to subsequent follow-up ultrasounds.

## Discussion

This study is unique in that it provides a prospective, in-depth analysis of parents’ responses to sonographers and what they needed from them. Parents who chose to continue their pregnancies with a LFD told us how meaningful their ultrasound experiences were. They came from diverse geographic, socio-economic, and educational backgrounds, and interviews included not only mothers, but also most fathers and one lesbian partner. The findings add significant depth to the sparse literature on parents’ experiences, particularly those of the father/partner, with their obstetric sonographers. The results also provide some clear guidelines for ultrasound providers.

Findings here bring to light the parents’ perspectives on ways that sonographers either blocked or facilitated their access to their baby within. Without access, parents felt powerless, and with access, they were able to visually be with their baby and have an updated assessment of the pregnancy. Seeing the baby on ultrasound was particularly important for the father or partner who was not physically able to feel the baby. To our knowledge, this finding has not been reported elsewhere.

Parents told us that their expectation of normalcy was shattered by devastating news during ultrasound. Their shock and grief, described by many previous studies (Aite et al., 2011; Côté-Arsenault & Denney-Koelsch, 2011; Skreden et al., 2010) was confirmed here. When parental and sonographer goals and expectations were mismatched, conflict could ensue. The wishes of each family were also variable, making the sonographer’s tasks of evaluating the families’ needs and performing the technical tasks of the sonogram challenging. Moreover, institutional variability in policies about information sharing by sonographers sometimes impeded good communications with a family.

Parents were upset when they felt that knowledge about their baby was being withheld from them, an emotional response that is consistent with the power differential that exists between the provider and patient, as described in conflict theory (O’Leary, 2005). Sonographers could be more sensitive to this disparity if they were aware that they possessed knowledge that the parents wanted and needed, and avoided putting them in a vulnerable position. Our participants reported that silence did not shelter them from worry. When parents were given partial information, provided without sensitivity to the impact of that incomplete knowledge, their already significant burden was increased.

In contrast, parents preferred highly engaged sonographers who explained what they were seeing and allowed them to ask questions. When sonographers assessed the parents’ expectations, explained their professional role, and modified their communication to match the parents’ needs, the interaction felt beneficial and highly satisfying, even when difficult information was being relayed. Those who openly and willingly used their technology as a conduit between the parent and their baby were received the most positively.

The primary limitation of this study is the exclusion of parents who chose to terminate their pregnancy and those with multiple gestations. Their experiences almost certainly differ from those presented here. Generalizability is also limited by known variations in care, due to sonographer training, scope of practice, and experience with families with LFDs. Although we had some sample diversity, all were English speaking and the range of religious or cultural views was limited. The strengths of this study were high retention, with participation of 30 parents with long-term engagement, and enhanced trustworthiness through consistent interviewing methods and clarification of interpretations with participants.

Based on our findings, we recommend eight communication strategies for providers of obstetrical ultrasounds to reduce the burden on families with lethal fetal conditions (see Table 3).

**Table 3.** Communication Strategies for Prenatal Ultrasound.

Recommendation	Comments	Example Phrases
1. Assess family’s prior experience and expectations	Informs the sonographer about parent preconceptions or fears	“Have you had an ultrasound before? What kind of experience did you have?” “Tell me what you are hoping to learn from this ultrasound.”
2. Reduce the power differential	• Make good eye contact • Sit down if possible when communicating information to parents	
3. Provide pre-test counseling	• Explain what will happen at the ultrasound and how information will be shared • Ask parents how much they would like to know	“I’ll be looking at all parts of the baby. I may need to spend some extra time at certain areas if it’s hard to see everything clearly. If there is anything I’m concerned about, would you like me to tell you?”
4. Communicate throughout ultrasound within the scope of practice	It is acceptable to indicate concern for a possible problem, allowing families to be included in the information about their baby, without overreaching the scope of sonographers’ practice	“I’m having a hard time seeing this area. It may not be a problem, but I want you to know why I’m spending more time here. I’ll have the doctor look as well.”
5. Allow time with the baby	Use baby’s name Point out not just anomalies, but also the normal and beautiful parts of the baby	“Have you come up with a name for your baby?” Is it alright if I use it?
6. Continuity of care	• Maintain the same sonographer at each visit if possible • Sonographer should be well informed of the patient’s history • It may be helpful to change an ultrasound lab’s protocol for following patients with lethal anomalies to provide better continuity of care	
7. Reduce waiting time	Have urgent appointments available for diagnosing serious anomalies, to minimize the parents’ long wait for confirmation	
8. Write down the findings	Families may look up information incorrectly without proper guidance. Sonographers should consult with physicians to guide patients in finding the information they want and need through pamphlets, written information, and websites	

## Conclusion

Obstetrical sonographers have a valuable opportunity to enhance a family’s experience with a LFD through sensitive, skilled interactions that match parental needs and expectations. Ultrasounds are a treasured opportunity for these families to get to know and enjoy precious time with their baby. More research is needed on educational interventions to improve sonographer–patient communication in high-risk situations, and to evaluate institutional policies that determine sonographer practice.
